# Supplementary material for: Association between Maternal Origin, Pre-Pregnancy Body Mass Index and Caesarean Section: A Nation-Wide Registry Study
Source: Int J Environ Res Public Health. 2021 Jun 1;18(11):5938. doi: 10.3390/ijerph18115938 (PMC8197892; doi:10.3390/ijerph18115938)
Supplement: Supplementary file 1 [file ijerph-18-05938-s001.zip › Table S1_.pdf]

Table S1: Proportions of elective and emergency CS by maternal region of origin in strata of pre-pregnancy BMI, n=118 459. (Supplementary material)

Maternal region of origin	Mode of delivery		
	**Vaginal**	**Elective CS**	**Emergency CS**
**Underweight (BMI<18.5 kg/m²)**	**N=3922 (87.70%)**	**N=143 (3.20%)**	**N=407 (9.10%)**
Norway	1874 (88.65)	81 (3.83)	159 (7.52)
High income countries	407 (49.25)	15 (3.29)	34 (7.46)
Europe/ & Central Asia	664 (89.01)	24 (3.22)	58 (7.77)
Sub-Saharan Africa	255 (82.30)	9 (2.91)	45 (14.56)
North Africa & the Middle East	213 (87.30)	5 (2.05)	26 (10.66)
South Asia	105 (83.33)	1 (0.79)	20 (15.56)
Southeast Asia, East Asia & the Pacific	374 (85.58)	5 (1.14)	58 (13.27)
Latin America & the Caribbean	30 (75.00)	3 (7.50)	7 (17.50)
**Normal weight (BMI 18.5-24.9 kg/m²)**	**N=50 676 (85.1%)**	**N=1744 (2.9%)**	**N=7122 (12.0%)**
Norway	31043 (86.27)	1055 (2.93)	3884 (10.79)
High income countries	6624 (85.74)	240 (3.11)	862 (11.16)
Europe & Central Asia	6322 (84.71)	249 (3.34)	892 (11.95)
Sub-Saharan Africa	1309 (73.91)	31 (1.75)	431 (24.34)
North Africa & the Middle East	1872 (83.83)	71 (3.18)	290 (12.99)
South Asia	917 (83.67)	27 (2.46)	152 (13.87)
Southeast Asia, East Asia & the Pacific	2174 (79.63)	50 (1.83)	506 (18.53)
Latin America & the Caribbean	415 (76.71)	21 (3.88)	105 (19.41)
**Overweight/ Obese (BMI ≥25.0 kg/m²)**	**N=21 304 (77.9)**	**N=939 (3.4)**	**N=5115 (18.7)**
Norway	15 156 (79.03)	655 (3.42)	3367 (17.56)
High income countries	2495 (78.04)	95 (2.97)	607 (18.99)
Europe & Central Asia	1542 (77.22)	98 (4.91)	357 (17.88)
Sub-Saharan Africa	482 (62.92)	27 (3.52)	257 (33.55)
North Africa & the Middle East	740 (75.98)	25 (2.57)	209 (21.46)
South Asia	318 (70.20)	18 (3.97)	117 (25.83)
Southeast Asia, East Asia & the Pacific	444 (72.31)	16 (2.61)	154 (25.08)
Latin America & the Caribbean	127 (70.95)	5 (2.79)	47 (26.26)
**Missing BMI**	**N=22 345 (82.5)**	**N=990 (3.7)**	**N=3752 (13.85)**
Norway	13 994 (83.45)	623 (3.72)	2152 (12.83)
High income countries	2812 (82.78)	139 (4.09)	446 (13.13)
Europe & Central Asia	2259 (83.70)	105 (3.89)	335 (12.41)
Sub-Saharan Africa	789 (73.74)	22 (2.06)	259 (24.41)
North Africa & the Middle East	945 (82.10)	41 (3.56)	165 (14.34)
South Asia	487 (80.90)	12 (1.99)	103 (17.11)
Southeast Asia, East Asia & the Pacific	855 (76.82)	38 (3.41)	220 (19.77)
Latin America & the Caribbean	204 (71.33)	10 (3.50)	72 (25.17)
